# Ocular adverse events associated with antibody-drug conjugates: a comprehensive pharmacovigilance analysis

**DOI:** 10.3389/fimmu.2024.1495137

**Published:** 2024-12-17

**Authors:** Heng Chen, Gefei He, Juanjuan Huang, Lin Hu, Junlong Ma

**Affiliations:** ^1^ Department of Pharmacy, The First Hospital of Changsha, Changsha, Hunan, China; ^2^ Center of Clinical Pharmacology, Third Xiangya Hospital, Central South University, Changsha, Hunan, China

**Keywords:** antibody-drug conjugates, ocular adverse events, pharmacovigilance, FAERS, real-world study

## Abstract

**Introduction:**

Antibody-drug conjugates (ADCs) are increasingly utilized in patients with solid tumors and hematologic malignancies. However, the adverse ocular toxicity induced by ADCs has not been comprehensively evaluated in real-world clinical settings.

**Methods:**

Data from April 2019 to March 2024 based on the FDA Adverse Event Reporting System (FAERS) were extracted and analyzed. Disproportionality analysis was used to evaluate the association between ADCs and ocular adverse events (AEs). The median time to onset (TTO) of various ADCs was compared.

**Results:**

A comprehensive analysis identified 2,686 ocular AEs associated with ADCs. Among these, Tisotumab vedotin had the most positive signals at the preferred terms (PTs) level, followed by trastuzumab emtansine and enfortumab vedotin. In contrast, gemtuzumab ozogamicin demonstrated minimal ocular toxicity signals. Cluster analysis revealed that ADC-related ocular toxicities predominantly manifested as corneal disorders or ocular neuromuscular disorders. The median onset of ocular toxicity varied considerably, with enfortumab vedotin showing the earliest median onset at 12.5 days.

**Conclusions:**

Our study demonstrates the association between ADCs and ocular AEs based on real-world data, providing valuable guidance for clinicians when prescribing ADCs. And we found some important safety signals that have not been mentioned in the label or previous studies.

## 1 Introduction

Antibody-drug conjugates (ADCs) represent an innovative class of anticancer therapeutics, heralding a significant paradigm shift in the management of malignancies and finding extensive application across both hematologic and solid tumors (1). Owing to their remarkable therapeutic efficacy, ADCs are often lauded as “magic bullets” within the realm of cancer treatment (2, 3). Structurally, ADCs comprise three integral components: a monoclonal antibody, a cytotoxic payload, and a linker (4). The monoclonal antibody is engineered to target specific antigens on the surface of cancer cells, thereby facilitating the precise delivery of potent cytotoxic agents directly to the tumor, minimizing off-target effects.

To date, the U.S. Food and Drug Administration (FDA) has approved 15 ADCs, which are categorized based on the nature of their cytotoxic payloads: tubulin polymerization inhibitors (such as trastuzumab emtansine, enfortumab vedotin, brentuximab vedotin, polatuzumab vedotin, and tisotumab vedotin), DNA-damaging agents (such as gemtuzumab ozogamicin, inotuzumab ozogamicin, trastuzumab deruxtecan, sacituzumab govitecan, and loncastuximab tesirine), and bacterial toxins (moxetumomab pasudotox). A report released at the end of 2023 highlights the rapid expansion of the global ADC market, which surged from USD 1.4 billion in 2016 to USD 11.3 billion in 2023, underscoring the substantial market potential of these agents (5).

The growing significance of ADCs as a therapeutic modality for oncologists is further emphasized by their expanding utilization in clinical practice (6). Nevertheless, the broad spectrum of adverse events (AEs) associated with ADC therapy demands vigilant consideration (7–10), as these AEs can precipitate treatment delays or discontinuation, thereby potentially compromising patient outcomes. Among the various AEs, ocular toxicities are notably prevalent during ADC therapy (6, 11, 12), often affecting the cornea or ocular surface and manifesting as blurred vision, foreign-body sensation, and clinical signs such as corneal fluorescein staining pseudomicrocysts and conjunctivitis (13).

Notably, ADCs such as enfortumab vedotin and tisotumab vedotin have been linked to a high incidence of ocular toxicity in clinical trials, with enfortumab vedotin in particular carrying a warning for ocular disorders (14, 15). Reports indicate that the incidence of ocular toxicities with enfortumab vedotin can reach as high as 46%, with dry eye symptoms being the most commonly reported, affecting up to 36% of patients. In the innovaTV 204 trial (15), ocular AEs were documented in 53% of patients receiving tisotumab vedotin, predominantly of mild to moderate severity, with grade 1/2 conjunctivitis and dry eye being the most frequent. The pathogenesis of these ADC-related ocular AEs may be attributed to the ADCs’ unique molecular structure, the cytotoxic mechanism of its payload, or the expression of target antigens on both tumor and healthy ocular cells (16). However, the precise mechanisms remain elusive.

Given the inherent limitations of clinical trials in fully elucidating the safety profile of drugs, coupled with the complex mechanisms underpinning ADC-mediated ocular toxicity, a comprehensive analysis based on real-world surveillance data is imperative. This is especially pertinent for ADCs that have received accelerated approval, fast-track designation, or priority review. The FDA’s Adverse Event Reporting System (FAERS) provides a publicly accessible database for global post-marketing surveillance. Recent studies have validated the accuracy of well-constructed pharmacovigilance analyses leveraging FAERS data (17, 18). In this study, we endeavor to provide an exhaustive examination of ocular AEs associated with ADCs, offering valuable insights to inform clinical practice.

## 2 Methods

### 2.1 Data source and collection

The FAERS database, established to support the FDA’s post-market safety surveillance of pharmaceuticals and biologics, systematically aggregates AE and medication errors from healthcare professionals, patients, and pharmaceutical manufacturers globally since 1968. This retrospective study utilized and analyzed AE reports extracted from the FAERS database, spanning the period from the second quarter of 2019 to the first quarter of 2024 (June 2019 to March 2023). All AEs were coded using the Medical Dictionary for Regulatory Activities (MedDRA, version 26.1). The FAERS data comprises seven dataset categories: demographic and administrative information (DEMO), drug details (DRUG), adverse events (REAC), patient outcomes (OUTC), report sources (RPSR), therapy start and end dates (THER), and indications for use/diagnosis (INDI). These datasets can be linked through the “PRIMARYID” and “CASEID”. As this study is observational and relies on anonymized data, approval from an ethics committee was not required.

### 2.2 Data pre-processing and extraction

**Figure 1:**
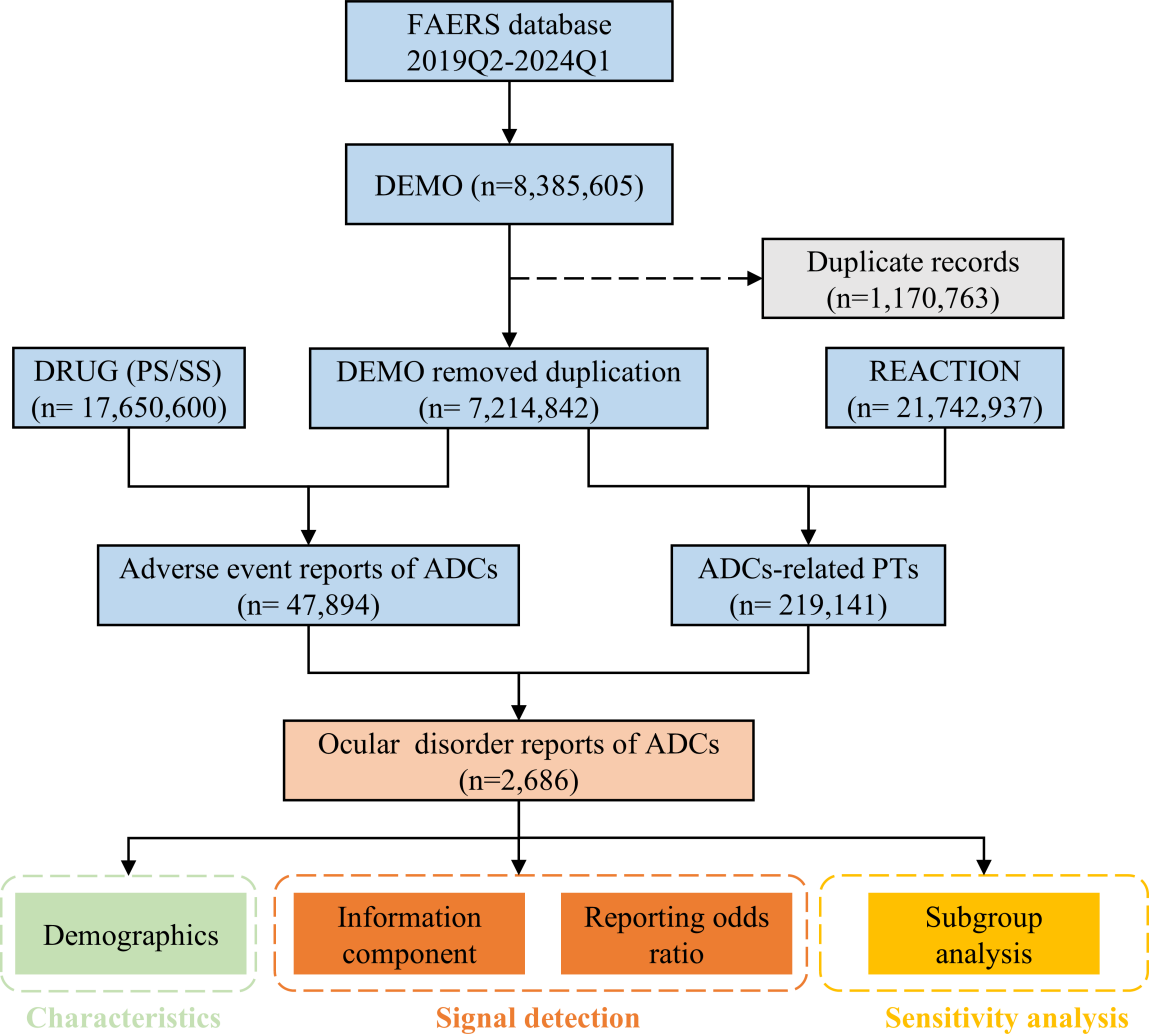
The flow diagram of the study. DEMO, demographic and administrative information; DRUG, drug information; REACTION, coded AEs.

A total of 8,385,605 reports were retrieved and subsequently imported into MySQL 8.0 (an database management system). Following FDA guidelines, we applied the variable matching method to address the duplicate reports (19). The primary process involved selecting the latest FDA_DT (date FDA received case) and the highest PRIMARYID when CASEID was identical, which resulted in the removal of 1,170,763 duplicate reports (**Figure 1**). We meticulously searched for 15 ADCs one by one, ultimately narrowing our focus to eight FDA-approved ADCs. These selected ADCs are gemtuzumab ozogamicin, brentuximab vedotin, trastuzumab emtansine, inotuzumab ozogamicin, polatuzumab vedotin, enfortumab vedotin, trastuzumab deruxtecan, and sacituzumab govitecan. To identify records of ocular events associated with ADCs, both generic and brand names were utilized. The role for AEs was assigned by the reporters with specific role codes—such as preferred suspect (PS), secondary suspect (SS), concomitant (C), and interacting (I). To enhance the accuracy of our analyses, we specifically included the reports with role code “PS” or “SS” (20).

In the FAERS database, AEs are classified using the Preferred Terms (PTs), which are specific descriptors for individual medical concepts such as signs, symptoms, and disease diagnoses. This hierarchical structure includes “high-level terms” (HLTs) and “high-level group terms” (HLGTs). At the highest level, HLGTs are organized into “system organ classes” (SOCs), which are categorized based on etiology, anatomical site of presentation, or intended therapeutic purpose. Ocular AEs related to the SOC of Eye disorders (Code: 10015919) were identified using the Medical Dictionary for Regulatory Activities (MedDRA) 26.1. Specifically, we also grouped AEs into HLGTs or broader categories known as Standardized MedDRA Queries (SMQs) to accurately describe specific disease conditions (21). The SMQs pertinent to ocular events in this study were “Conjunctival disorders”, “Corneal disorders”, “Periorbital and eyelid disorders”, “Retinal disorders”, “Optic nerve disorders”, and “Ocular infections”.

After implementing the deduplication and screening processes, a total of 2,686 unique ocular AE reports associated with ADCs were identified and subjected to further analysis.

### 2.3 Data mining and statistical analysis

Descriptive statistics were calculated to summarize patient demographics. Categorical variables were presented as frequencies and percentages, while continuous variables were summarized as medians with interquartile ranges.

A disproportionality analysis using a case/non-case approach was conducted to explore the potential association between ADCs and reports of ocular AEs. This analysis was performed at both the PT, HLGT, and SMQ levels to determine if the proportion of a reported event of interest for a specific drug differed from the proportion of the same AEs in the control drug group (22). In this study, disproportionality was assessed using the Bayesian (information component, IC) (23) and the Frequentist (reporting odds ratio, ROR) (24–26). A signal was defined when the lower bound of the 95% CI for IC (IC_025_) was greater than 0, or when the lower bound of the 95% confidence interval (CI) for ROR (ROR_025_) exceeded 1, indicating that the drug-AE pair of interest was reported more frequently compared to the control group. A statistical shrinkage transformation was applied to ensure more robust results, with the specific formulas for the transformed ROR and IC detailed in **Supplementary Table S1**. The time to onset, defined as the duration between ADCs administration and the occurrence of AEs, was also assessed. The median number of days, along with the corresponding interquartile range, was depicted. The parametric distribution model that yielded the best fit according to the goodness-of-fit test was selected (27). A sensitivity analysis was conducted to evaluate the impact of concomitant medications on the outcomes. Drugs known to be associated with ocular AEs were identified and subsequently excluded from the analysis.

The Modified Bradford Hill Criteria, commonly employed in epidemiology, were utilized to evaluate the potential relationships among various available evidence (27). This assessment considered key factors such as biological plausibility, strength of association, consistency, specificity, coherence, and analogy.

All data analyses were conducted independently by two authors. Data extraction was performed using MySQL 8.0, and statistical analyses were carried out using Python 3.10 and SPSS 27.0.

## 3 Results

### 3.1 Clinical characteristics

A total of 2,686 ocular AEs associated with ADCs were identified from the FAERS database, spanning from the second quarter of 2019 to the first quarter of 2024. The clinical characteristics of the patients involved are detailed in **Table 1**. It was observed that brentuximab vedotin, enfortumab vedotin, gemtuzumab ozogamicin, inotuzumab ozogamicin, and polatuzumab vedotin were predominantly reported in male patients. In contrast, sacituzumab govitecan, tisotumab vedotin, trastuzumab deruxtecan, and trastuzumab emtansine were overwhelmingly reported in female patients. Notably, 66.7% of patients reporting ocular toxicity associated with inotuzumab ozogamicin were under 18 years of age, with a median age of 7 years. Patients receiving enfortumab vedotin, polatuzumab vedotin, and sacituzumab govitecan were predominantly over 45 years old, with median ages of 73 (IQR: 65-79), 74 (IQR: 65-74), and 58 (IQR: 52.25-64.25) years, respectively. Other ADCs were reported in patients aged predominantly between 18 and 64 years. Geographic trends indicated that reports for brentuximab vedotin, inotuzumab ozogamicin, polatuzumab vedotin, and trastuzumab emtansine were mainly from Europe, whereas other ADCs were primarily reported from the United States. Regardless of the outcomes, ADCs were more likely to result in initial or prolonged hospitalization, except sacituzumab govitecan and trastuzumab deruxtecan, which were more frequently associated with fatal outcomes. Mortality rates varied across ADCs, with relatively high proportions (16.1%–40%) observed for sacituzumab govitecan, trastuzumab emtansine, trastuzumab deruxtecan, and inotuzumab ozogamicin.

**Table 1:** Demographic information on ocular toxicity with ADCs.

	brentuximab vedotin	enfortumab vedotin	gemtuzumab ozogamicin	inotuzumab ozogamicin	polatuzumab vedotin	sacituzumab govitecan	tisotumab vedotin	trastuzumab deruxtecan	trastuzumab emtansine
**Total Cases**	60	115	12	10	50	40	103	118	292
Gender									
Data available	53	111	11	9	37	38	79	99	248
Female	24(45.3%)	33(29.7%)	3(27.3%)	4(44.4%)	16(43.2%)	38(100%)	77(97.5%)	91(91.9%)	246(99.2%)
Male	29(54.7%)	78(70.3%)	8(72.7%)	5(55.6%)	21(56.8%)	0(0%)	2(2.5%)	8(8.1%)	2(0.8%)
Age									
Data available	45	83	7	9	30	24	29	61	211
<18	0(0%)	0(0%)	0(0%)	6(66.7%)	0(0%)	0(0%)	0(0%)	1(1.6%)	1(0.5%)
18–44	18(40.0%)	1(1.2%)	4(57.1%)	0(0%)	1(3.3%)	2(8.3%)	10(34.5%)	22(36.1%)	51(24.2%)
45–64	17(37.8%)	14(16.9%)	3(42.9%)	1(11.1%)	7(23.3%)	16(66.7%)	15(55.2%)	29(47.5%)	126(59.7%)
65–74	6(13.3%)	29(34.9%)	0(0%)	2 (22.2%)	15(50%)	4(16.7%)	2(6.9%)	5(8.2%)	29(13.7%)
>74	4(8.9%)	39(47.0%)	0(0%)	0(0%)	7 (23.3%)	2(8.3%)	1(3.4%)	4(6.6%)	4(1.9%)
Median (IQR)	46(43-61)	73(65-79)	44(30-53.5)	7(3-48)	74(65-74)	58(52.25-64.25)	47(41-59)	51(33-60)	57(46-61)
Reported countries (Top 3)									
1	FR 27(45%)	US 64(55.7%)	US 4(33.3%)	FR 4(40%)	DE 11(22%)	US 12(30%)	US 91(88.3%)	US 50(22%)	GB 99(33.9%)
2	US 10(16.7%)	JP 36(31.3%)	ES 2 (16.7%)	PL 2(20%)	HR 9(18%)	FR 10(25%)	ES 6(5.8%)	FR 21(18%)	CA 63(21.6%)
3	IL 4(6.7%)	CA 4(3.5%)	GB 1(8.3%)	CN 1(10%)	IT 8(16%)	CA 7(17.5%)	IT 8(2.9%)	CA 17(16%)	US 39(13.4%)
Outcomes									
Data available	54	90	12	10	50	31	42	78	265
Hospitalized	9(16.7%)	21(23.3%)	7(58.3%)	6(60%)	8(16%)	4(12.9%)	4(9.5%)	15(19.2%)	89(33.6%)
Disabled	9(16.7%)	2(2.2%)	0(0%)	0(0%)	1(2%)	0(0%)	2(4.8%)	5(6.4%)	11(4.2%)
Congenital Anomaly	0(0%)	0(0%)	0(0%)	0(0%)	0(0%)	0(0%)	0(0%)	0(0%)	6(2.3%)
Life threating	6(11.1%)	1(1.1%)	0(0%)	0(0%)	0(0%)	0(0%)	1(2.4%)	0(0%)	6(2.3%)
Died	1(1.9%)	7(7.8%)	0(0%)	4(40%)	5(0%)	5(16.1%)	0(0%)	19(24.4%)	47(17.7%)
Other outcomes	29(53.7%)	59(65.6%)	5(41.7%)	0(0%)	36(72%)	22(71%)	35(83.3%)	39(50%)	106(40%)

FR, France; US, United States; DE, Germany; GB, United Kingdom; JP, Japan; ES, Spain; PL, Poland; HR, Croatia; CA, Canada; IL: Israel; CN, China; IT, Italy; IQR, interquartile range.

### 3.2 Category of ADC-associated ocular AEs

**Figure 2:**
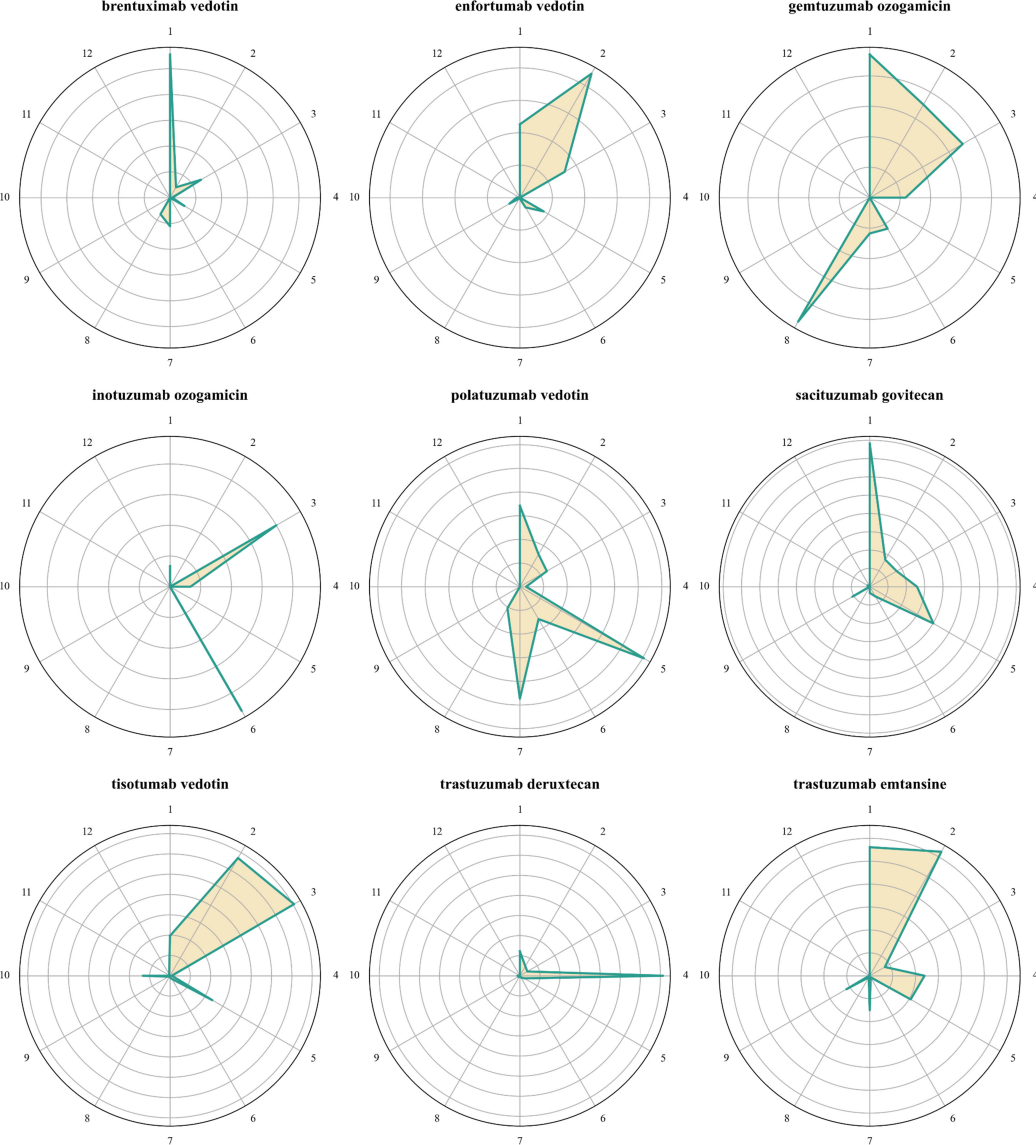
Radar plots of the distribution of ocular AE at the HLGT level. 1. Vision disorders; 2. Eye disorders NEC; 3. Ocular infections, irritations and inflammations; 4. Ocular neuromuscular disorders; 5. Anterior eye structural change, deposit and degeneration; 6. Ocular hemorrhages and vascular disorders NEC; 7. Ocular structural change, deposit and degeneration NEC; 8. Retina, choroid and vitreous hemorrhages and vascular disorders; 9. Ocular sensory symptoms NEC; 10. Ocular injuries; 11. Glaucoma and ocular hypertension; 12. Ocular neoplasms.

The proportion of ADC-associated ocular AEs relative to the total AEs for each drug is illustrated in **Supplementary Figure S1**. Notably, ocular AEs associated with tisotumab vedotin constituted 21.29% of all AEs reported for this drug. In comparison, the percentages of ocular AEs associated with enfortumab vedotin, trastuzumab emtansine, and trastuzumab deruxtecan were 1.76%, 1.71%, and 1.49%, respectively. **Figure 2** presents the distribution of ocular AEs at the HLGT level. Brentuximab vedotin and trastuzumab deruxtecan were primarily associated with vision disorders and ocular neuromuscular disorders, respectively. Enfortumab vedotin and tisotumab vedotin were mainly linked to eye disorders NEC and ocular infections, irritations, and inflammations. Additionally, enfortumab vedotin and tisotumab vedotin were associated with vision disorders and anterior eye structural change, deposit and degeneration, respectively. The ocular toxicities of gemtuzumab ozogamicin were predominantly vision disorders, ocular infections, irritations and inflammations, and retinal, choroid, and vitreous hemorrhages and vascular disorders. Inotuzumab ozogamicin was mainly associated with ocular infections, irritations and inflammations, and ocular hemorrhages and vascular disorders NEC. Polatuzumab vedotin and sacituzumab govitecan were predominantly linked to vision disorders and anterior eye structural changes, deposits, and degeneration, with polatuzumab vedotin also associated with ocular structural changes, deposits, and degeneration NEC. Trastuzumab emtansine was primarily linked to vision disorders and eye disorders NEC.

### 3.3 Specific PTs on the ocular toxicity of ADCs

**Figure 3:**
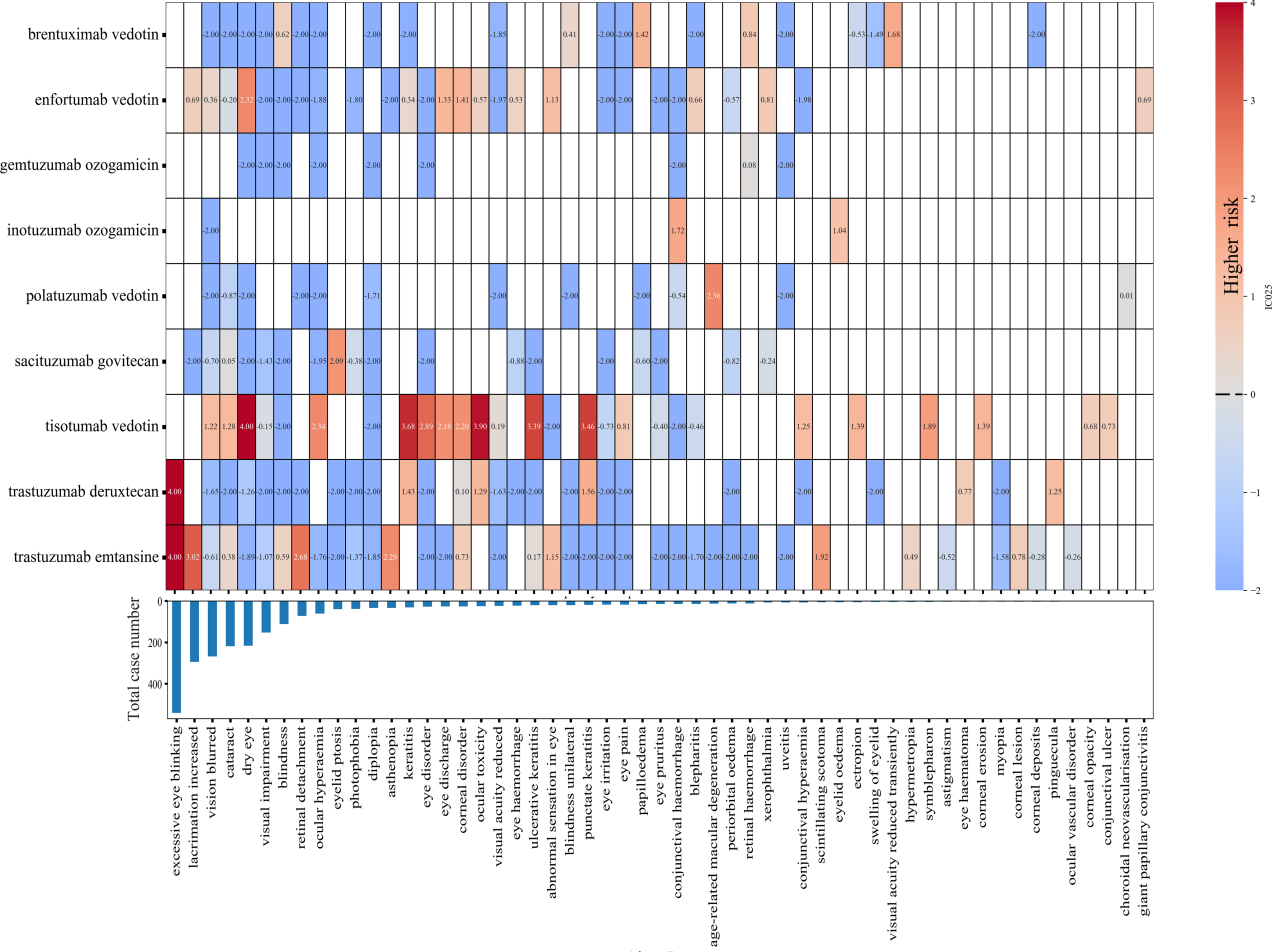
The heatmap showing the associations between ADCs and ocular adverse events at the preferred term level.

At the PTs level, all ADC-associated ocular AE reports were ranked by cumulative frequency (**Figure 3**). Tisotumab vedotin exhibited the highest number of positive signals (19 positive signals), followed by trastuzumab emtansine (12 positive signals) and enfortumab vedotin (12 positive signals). Tisotumab vedotin was strongly associated with specific PTs, including the dry eye (IC_025_ = 4.39), ocular toxicity (IC_025_ = 3.90), keratitis (IC_025_ = 3.68), punctate keratitis (IC_025_ = 3.46), and ulcerative keratitis (IC_025_ = 3.39). Trastuzumab deruxtecan showed the strongest correlation with excessive eye blinking (IC_025_ = 8.28), while trastuzumab emtansine also had a notable association with excessive eye blinking (IC_025_ = 5.89) and increased lacrimation (IC_025_ = 3.02). Conversely, gemtuzumab ozogamicin had the weakest correlation with ocular AEs, with IC_025_ values below 0 for all PTs except for retinal hemorrhage (IC_025_ = 0.08).

### 3.4 Association of ADCs with selected ocular disorders

**Figure 4:**
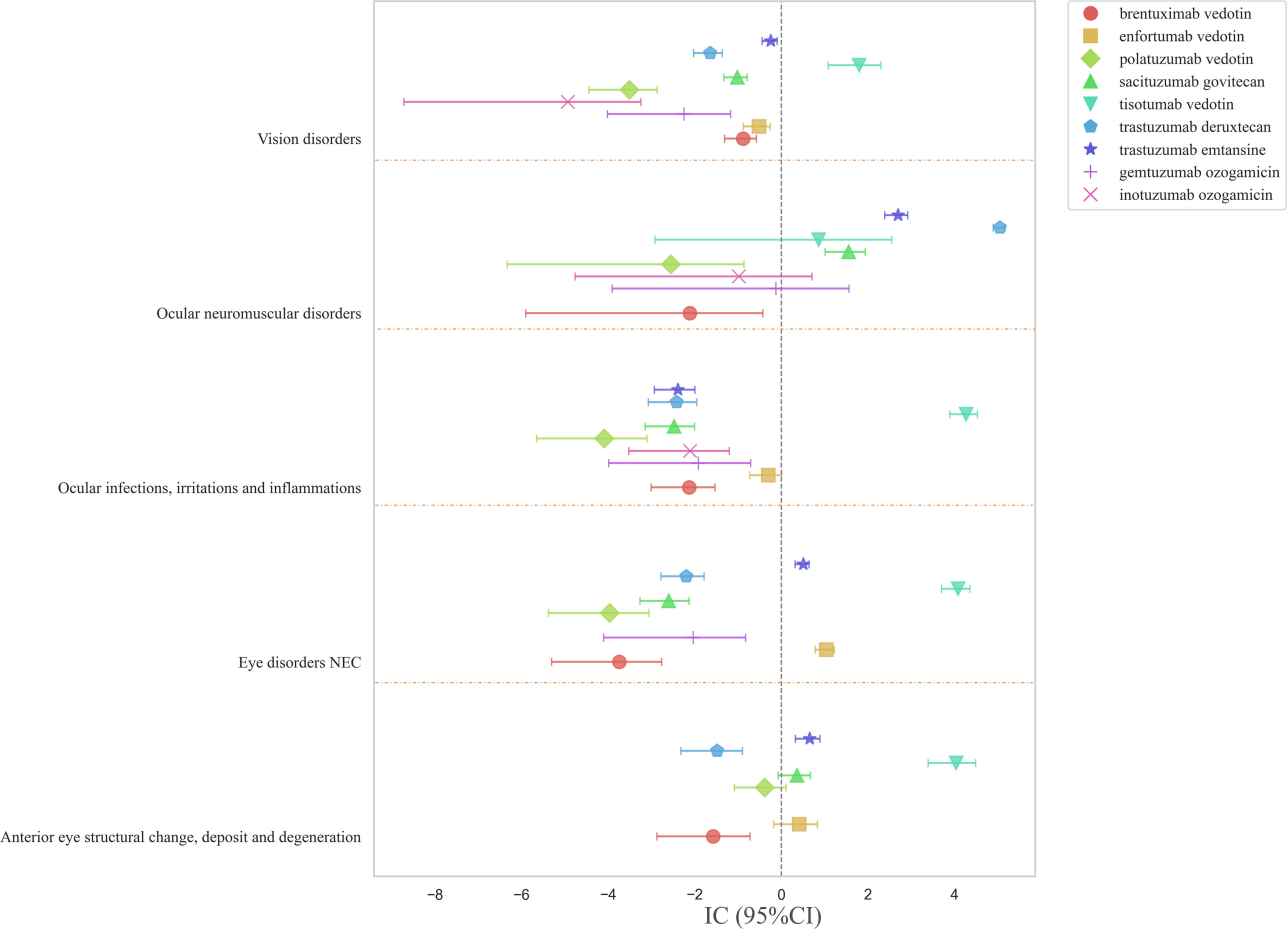
Forest plots of ICs under HLGT classification of various ADCs.

Based on the results in **Figure 2**, the top five ocular disorders were identified at the HLGT level, and the associations of different ADCs with these disorders were analyzed (**Figure 4**). ADCs were more commonly associated with ocular neuromuscular disorders and eye disorders NEC, each involving three drugs. Trastuzumab deruxtecan exhibited the strongest correlation with ocular neuromuscular disorders (IC: 5.05; 95%CI: 4.89-5.17). Tisotumab vedotin had the broadest range of involvement, covering four types of ocular disorders with strong correlations. In contrast, brentuximab vedotin, gemtuzumab ozogamicin, inotuzumab ozogamicin, and polatuzumab vedotin did not show significant associations with these ocular disorders.

At the SMQ level, ADC-associated ocular toxicities were most concentrated in corneal disorders, involving four drugs: tisotumab vedotin, trastuzumab deruxtecan, trastuzumab emtansine, and enfortumab vedotin (**Supplementary Table S2**). This was followed by conjunctival disorders and ocular infections, each involving three drugs. Tisotumab vedotin demonstrated the widest range of ocular toxicities, covering four types of ocular disorders with the highest risk signals, particularly for corneal disorders (IC: 6.06; 95%CI: 5.61-6.39) and conjunctival disorders (IC: 5.40; 95%CI: 5.07-5.64). On the other hand, gemtuzumab ozogamicin, inotuzumab ozogamicin, and polatuzumab vedotin did not exhibit significant risk associations with any ocular disorders.

In populations where only ADCs were used, ocular toxicity associated with ADCs was analyzed, and the results are presented in **Table 2**. It was found that enfortumab vedotin, tisotumab vedotin, trastuzumab deruxtecan, and trastuzumab emtansine all exhibited positive signals for ocular toxicity, with tisotumab vedotin demonstrating the strongest signal (IC: 4.11; 95%CI: 3.89-4.27).

**Table 2:** Disproportionality analysis of reports involving ADCs with all ocular disorders.

Drug	Cases	Non-cases	IC (95% CI)	ROR (95% CI)
brentuximab vedotin	28	19,511	-3.03 (-3.66, -2.58)	0.12 (0.08, 0.18)
enfortumab vedotin	376	21,030	0.56 (0.39, 0.69)	1.48 (1.32, 1.65)
gemtuzumab ozogamicin	17	3,662	-1.34 (-2.15, -0.77)	0.40 (0.25, 0.64)
inotuzumab ozogamicin	15	7,970	-2.62 (-3.49, -2.02)	0.16 (0.10, 0.27)
polatuzumab vedotin	76	26,881	-2.07 (-2.45, -1.80)	0.24 (0.19, 0.30)
sacituzumab govitecan	294	40,695	-0.73 (-0.92, -0.59)	0.60 (0.53, 0.68)
tisotumab vedotin	224	828	4.11 (3.89, 4.27)	17.26 (14.81, 20.11)
trastuzumab deruxtecan	607	40,203	0.32 (0.19, 0.42)	1.25 (1.14, 1.37)
trastuzumab emtansine	969	55,755	0.52 (0.42, 0.60)	1.44 (1.33, 1.56)

To address the impact of potential confounding factors, such as the use of concomitant medications, we provided an overview of the ocular toxicity risks associated with the top 10 co-administered drugs in **Supplementary Table S3**. Utilizing information from FDA labels, we identified that frequently co-prescribed drugs, including docetaxel (18.6%), dexamethasone (16.4%), and ondansetron (9.2%), might contribute to ocular AEs. As a result, these three drugs were categorized as confounder medications. Sensitivity analyses were performed by excluding cases involving these confounder drugs (**Supplementary Figure S2**). The largest reduction in case numbers was observed with trastuzumab emtansine, while the influence of other drugs on confounding effects was minimal.

### 3.5 Time to onset of ADCs with high ocular toxicity risk

Based on previous findings, four ADCs with relatively higher risk signals for ocular toxicity were selected for analysis of onset times, with results depicted in **Supplementary Figure S3**. Enfortumab vedotin had the earliest onset with a median of 12.5 days (IQR: 6.5-28.5 days), while trastuzumab deruxtecan had the latest onset with a median of 93.5 days (IQR: 43.5-179.5 days). Tisotumab vedotin and trastuzumab emtansine had median onset times of 41.5 days (IQR: 7.5-95 days) and 37.5 days (IQR: 2.5-112.5 days), respectively.

### 3.6 Causal relationship global assessment

Overall, the Bradford Hill Criteria were satisfied, as evidenced by the strength and consistency of disproportionality analyses, the observed temporal relationship, and biological plausibility. These findings support a probable causal association between ADCs and monoclonal ocular AEs (**Supplementary Table S4**).

## 4 Discussion

ADCs have been widely employed in the treatment of various solid tumors and hematologic malignancies due to their superior efficacy. However, the safety concerns associated with ADCs in real-world applications should not be overlooked, particularly given the limitations of short follow-up periods, small sample sizes, and stringent patient selection criteria in the clinical trial phase. Although case reports of ocular toxicity related to ADCs exist, a systematic evaluation of this toxicity is lacking. This study provides a comprehensive analysis of the ocular toxicity of ADCs using real-world case reports from the FAERS database in a post-marketing setting, aiming to provide direct evidence to inform clinical decision-making regarding ADC therapy. To our knowledge, this study represents the most extensive and exhaustive characterization of ADC-related ocular AEs to date.

According to data from the FAERS database, the patients reporting ocular toxicity related to inotuzumab ozogamicin were predominantly pediatric. Conversely, Brentuximab vedotin and gemtuzumab ozogamicin, both approved for pediatric use, did not show reports of ocular toxicity in minors. Thus, in clinical practice, attention should be given to the risk of ocular toxicity in pediatric patients treated with inotuzumab ozogamicin. Overall, our analysis indicated that mortality accounted for over 10% of all ocular AE records associated with ADCs, a rate higher than that of other drug-related ocular AEs (28–30), underscoring the significant impact of ADC-related ocular AEs on patient mortality. Additionally, enfortumab vedotin, one of the ADCs with a higher risk of ocular toxicity, was associated with earlier onset of symptoms, possibly due to its higher dosing frequency. While it is administered on a standard 21-day cycle, similar to other ADCs, the increased dosing frequency of enfortumab vedotin may contribute to greater drug accumulation, potentially precipitating a more rapid onset of ocular complications. In contrast, trastuzumab deruxtecan had a median onset time of 93.5 days, indicating the need for long-term follow-up in patients undergoing prolonged treatment, with vigilance for potential late-onset ocular toxicity.

At the SMQ level, our findings suggest a higher risk of conjunctival diseases, corneal diseases, and ocular infections associated with ADCs. This may be attributed to the expression of ADC-related target proteins in ocular tissues, particularly the conjunctiva and cornea (31, 32). Furthermore, we identified several novel AEs at the PT level: tisotumab vedotin was strongly linked to cataracts; enfortumab vedotin was associated with blepharitis and eye hemorrhage; polatuzumab vedotin exhibited a strong association with age-related macular degeneration. It is noteworthy that the prescribing information for brentuximab vedotin, gemtuzumab ozogamicin, inotuzumab ozogamicin, and sacituzumab govitecan does not mention ocular AEs, yet these ADCs were reported to have a lower risk of ocular toxicity in our study. However, some potential drug-related ocular AE signals were uncovered. Brentuximab vedotin demonstrated a strong correlation with visual acuity reduced transiently and papilledema. Inotuzumab ozogamicin may induce conjunctival hemorrhage and eyelid oedema, while sacituzumab govitecan was strongly associated with eyelid ptosis. Gemtuzumab ozogamicin did not report significant ocular toxicity signals. Healthcare providers need to be aware of these potential ocular toxicities to promptly detect and manage ocular complications in patients.

Ocular toxicity is a particularly notable AE during treatment with tisotumab vedotin, extensively reported in clinical trials, manifesting as conjunctivitis, dry eye, and keratitis (33, 34). Additionally, according to the prescribing information and clinical trial data, polatuzumab vedotin, enfortumab vedotin, sacituzumab govitecan, trastuzumab deruxtecan, and trastuzumab emtansine have been associated with dry eye, blurred vision, conjunctivitis, and keratitis (35–37). Except for polatuzumab vedotin, which has a prescribing note mentioning possible blurred vision, ocular toxicities have not been widely reported for other ADCs targeting hematologic malignancies. In our study, compared to ADCs targeting hematologic malignancies, those targeting solid tumors were more likely to induce ocular AEs, with a higher prevalence of ocular diseases at HLGT or SMQ level and stronger associations with multiple AEs at PT level. The results remained consistent when the analysis was limited to patients treated with ADCs. The difference in ocular toxicity between ADCs targeting solid tumors and those targeting hematologic malignancies may be attributed to differences in the molecular structure of the drugs and the cytotoxic mechanisms of the payload. In the sensitivity analysis, ocular AEs associated with ADCs, with the exception of trastuzumab emtansine, appeared largely unaffected by concomitant medications. For trastuzumab emtansine, our prior research demonstrated a persistent and significant association even after adjusting for confounding factors (38).

The mechanisms of ADC-induced ocular toxicity can be categorized into off-target and on-target toxicities. Off-target toxicity is often mediated by the cytotoxic payload (39), while on-target toxicity arises when the drug interacts with its intended receptor. Hematologic toxicity, the most common serious AE associated with ADCs, may be triggered by premature release of the cytotoxic payload, similar to traditional chemotherapy drugs targeting rapidly proliferating cells (1, 40). Ocular toxicity has also been reported in maytansinoid- and monomethyl auristatin F (MMAF)-containing ADCs targeting antigens not expressed in ocular tissues, such as CD19, folate receptor alpha, and mesothelin (41–43). However, our study identified significant variability in ocular toxicity risks among monomethyl auristatin E (MMAE)-containing ADCs, with tisotumab vedotin (targeting TF) and enfortumab vedotin (targeting Nectin-4) showing higher risks. Similarly, HER2-targeting ADCs also demonstrated elevated ocular toxicity risks. Thus, ocular toxicity may be influenced not only by the cytotoxic payload but also by differences in the targeting antibodies. Preclinical studies have shown that TF is expressed in human conjunctiva and is closely associated with the occurrence and progression of pterygium and age-related macular degeneration (44, 45). Studies further demonstrate that HER2 and Nectin-4, proteins expressed in conjunctival and corneal epithelial cells, play pivotal roles in signaling pathways essential for maintaining ocular health, notably the ERK and PI3K-AKT cascades (31, 46–48). These findings suggest that both off-target effects mediated by cytotoxic payloads and on-target mechanisms involving TF, HER2, and Nectin-4 contribute to the observed ocular toxicity in ADCs.

This study offered in-depth insights into ocular AEs associated with the use of ADCs by analyzing data from the FAERS database and using the adjusted Bradford Hill criteria. However, several inherent limitations of pharmacovigilance studies are acknowledged. Firstly, the FAERS database, which relies on spontaneous reporting, may be subject to various reporting biases. Secondly, disproportionality analysis cannot quantify risk or establish causality, as the IC or ROR merely indicates an increased likelihood of AE reporting. Thirdly, cases in the FAERS database may lack complete information, including dosage, complications, and other relevant factors. Therefore, further research is necessary to validate our findings. Despite these limitations, the FAERS database remains a valuable resource with a substantial sample size in pharmacovigilance studies.

## 5 Conclusion

By leveraging real-world data from the FAERS database, this study provides a comprehensive analysis of ocular AEs associated with ADCs. Beyond the well-documented ocular AEs linked to ADCs, we identified significant novel ocular AEs. Moreover, our findings indicate that the relative risk of ocular toxicity varies among different types of ADCs, potentially due to the differential expression of their targets within ocular tissues. In summary, our results may enhance awareness of ADC-related ocular toxicities and support healthcare professionals in mitigating the risk. Further rigorously designed studies are essential to confirm these findings and provide more robust evidence.

## Data Availability

The raw data supporting the conclusions of this article will be made available by the authors, without undue reservation.
