# Supplementary material for: Ocular adverse events associated with antibody-drug conjugates: a comprehensive pharmacovigilance analysis
Source: Front Immunol. 2024 Dec 17;15:1495137. doi: 10.3389/fimmu.2024.1495137 (PMC11685049; doi:10.3389/fimmu.2024.1495137)
Supplement: Supplementary file 1 [file Table1.docx]

**Table S1.** Major algorithms used for pharmacovigilance analysis.

| **Algorithms** | **Equation** | **Criteria** |
| --- | --- | --- |
| ROR | ROR=ad/b/c | ROR_025_ > 1 |
|  | 95%CI=e^ln(ROR)±1.96(1/a+1/b+1/c+1/d)^0.5^ |  |
| IC | IC=log_2_a(a+b+c+d)(a+c)(a+b) | IC_025_ > 0 |
|  | 95%CI= E(IC) ± 2V(IC)^0.5 |  |

Abbreviations: a, number of reports containing both the target drug and target adverse event; b, number of reports containing other adverse event of the target drug; c, number of reports containing the target adverse event of other drugs; d, number of reports containing other drugs and other adverse event. 95%CI, 95% confidence interval; N, the number of reports; E(IC), the IC expectations; V(IC), the variance of IC.

**Table S2.** Signal detection for ADC-associated ocular adverse events at SMQ level.

| **Drug** | **Ocular disorders** | **IC (95% CI)** | **ROR (95% CI)** |
| --- | --- | --- | --- |
| gemtuzumab ozogamicin | Conjunctival disorders | -0.33 (-1.89, 0.66) | 0.80 (0.33, 1.91) |
| brentuximab vedotin | Conjunctival disorders | -2.20 (-3.51, -1.35) | 0.22 (0.10, 0.46) |
| trastuzumab emtansine | Conjunctival disorders | 0.73 (0.47, 0.91) | 1.66 (1.42, 1.93) |
| inotuzumab ozogamicin | Conjunctival disorders | -0.94 (-2.24, -0.09) | 0.52 (0.25, 1.09) |
| polatuzumab vedotin | Conjunctival disorders | -1.28 (-2.05, -0.75) | 0.41 (0.26, 0.64) |
| enfortumab vedotin | Conjunctival disorders | 2.10 (1.83, 2.28) | 4.27 (3.66, 4.99) |
| trastuzumab deruxtecan | Conjunctival disorders | -0.56 (-1.04, -0.22) | 0.68 (0.51, 0.90) |
| sacituzumab govitecan | Conjunctival disorders | -2.52 (-3.50, -1.86) | 0.17 (0.10, 0.31) |
| tisotumab vedotin | Conjunctival disorders | 5.40 (5.07, 5.64) | 42.22 (34.29, 51.99) |
| brentuximab vedotin | Corneal disorders | -1.28 (-3.87, 0.11) | 0.41 (0.10, 1.65) |
| trastuzumab emtansine | Corneal disorders | 0.87 (0.26, 1.30) | 1.83 (1.28, 2.62) |
| enfortumab vedotin | Corneal disorders | 1.03 (0.09, 1.67) | 2.04 (1.19, 3.52) |
| trastuzumab deruxtecan | Corneal disorders | 1.38 (0.78, 1.80) | 2.60 (1.82, 3.69) |
| sacituzumab govitecan | Corneal disorders | -3.02 (-6.81, -1.34) | 0.12 (0.02, 0.87) |
| tisotumab vedotin | Corneal disorders | 6.06 (5.61, 6.39) | 66.88 (50.68, 88.26) |
| brentuximab vedotin | Periorbital and eyelid disorders | -0.99 (-2.06, -0.26) | 0.50 (0.27, 0.94) |
| trastuzumab emtansine | Periorbital and eyelid disorders | -1.17 (-1.82, -0.70) | 0.45 (0.30, 0.65) |
| inotuzumab ozogamicin | Periorbital and eyelid disorders | -0.44 (-1.85, 0.47) | 0.74 (0.33, 1.64) |
| enfortumab vedotin | Periorbital and eyelid disorders | 0.37 (-0.25, 0.81) | 1.30 (0.90, 1.87) |
| trastuzumab deruxtecan | Periorbital and eyelid disorders | -2.18 (-3.32, -1.41) | 0.22 (0.12, 0.43) |
| sacituzumab govitecan | Periorbital and eyelid disorders | 0.46 (0.03, 0.77) | 1.38 (1.07, 1.78) |
| tisotumab vedotin | Periorbital and eyelid disorders | 3.61 (2.84, 4.15) | 12.23 (7.77, 19.26) |
| gemtuzumab ozogamicin | Retinal disorders | 0.07 (-1.70, 1.15) | 1.05 (0.39, 2.79) |
| brentuximab vedotin | Retinal disorders | 1.26 (0.79, 1.60) | 2.40 (1.81, 3.17) |
| trastuzumab emtansine | Retinal disorders | 0.54 (0.18, 0.79) | 1.45 (1.17, 1.79) |
| polatuzumab vedotin | Retinal disorders | -0.27 (-0.97, 0.22) | 0.83 (0.55, 1.25) |
| enfortumab vedotin | Retinal disorders | -2.33 (-4.09, -1.25) | 0.20 (0.07, 0.53) |
| trastuzumab deruxtecan | Retinal disorders | -2.32 (-3.54, -1.52) | 0.20 (0.10, 0.40) |
| tisotumab vedotin | Retinal disorders | -0.08 (-3.86, 1.61) | 0.95 (0.13, 6.73) |
| gemtuzumab ozogamicin | Optic nerve disorders | 0.03 (-3.75, 1.72) | 1.02 (0.14, 7.25) |
| brentuximab vedotin | Optic nerve disorders | 0.89 (-0.18, 1.62) | 1.86 (1.00, 3.46) |
| trastuzumab emtansine | Optic nerve disorders | -1.78 (-3.55, -0.70) | 0.29 (0.11, 0.78) |
| polatuzumab vedotin | Optic nerve disorders | -0.23 (-1.64, 0.68) | 0.85 (0.38, 1.90) |
| enfortumab vedotin | Optic nerve disorders | -2.03 (-5.82, -0.35) | 0.24 (0.03, 1.73) |
| trastuzumab deruxtecan | Optic nerve disorders | -2.91 (-6.69, -1.22) | 0.13 (0.02, 0.95) |
| sacituzumab govitecan | Optic nerve disorders | -0.59 (-1.89, 0.26) | 0.66 (0.32, 1.39) |
| gemtuzumab ozogamicin | Ocular infections | 0.93 (-0.84, 2.01) | 1.90 (0.71, 5.08) |
| brentuximab vedotin | Ocular infections | 2.10 (1.60, 2.46) | 4.28 (3.18, 5.76) |
| trastuzumab emtansine | Ocular infections | -2.70 (-4.46, -1.62) | 0.15 (0.06, 0.41) |
| inotuzumab ozogamicin | Ocular infections | -1.60 (-5.38, 0.09) | 0.33 (0.05, 2.34) |
| polatuzumab vedotin | Ocular infections | 0.13 (-0.74, 0.73) | 1.10 (0.66, 1.82) |
| enfortumab vedotin | Ocular infections | 0.99 (0.27, 1.49) | 1.98 (1.31, 3.01) |
| trastuzumab deruxtecan | Ocular infections | -1.70 (-3.12, -0.79) | 0.31 (0.14, 0.68) |
| tisotumab vedotin | Ocular infections | 3.48 (2.45, 4.17) | 11.13 (6.15, 20.18) |

**Table S3**. Top 10 concomitant drugs with ADCs in ocular cases

| **Concomitant drugs** | **N (%)** | **Ocular AEs (FDA Label information)** |
| --- | --- | --- |
| Docetaxel | 142 (18.6%) | Cystoid macular edema (CME) has been reported in patients treated with Docetaxel Injection. Patients with impaired vision should undergo a prompt and comprehensive ophthalmologic examination. If CME is diagnosed, Docetaxel Injection treatment should be discontinued and appropriate treatment initiated. Alternative non-taxane cancer treatment should be considered. |
| Dexamethasone | 125 (16.4%) | Use of corticosteroids may produce posterior subcapsular cataracts, glaucoma with possible damage to the optic nerves, and may enhance the establishment of secondary ocular infections due to bacteria, fungi, or viruses. Consider referral to an ophthalmologist for patients who develop ocular symptoms or use corticosteroid-containing products for more than 6 weeks. The use of oral corticosteroids is not recommended in the treatment of optic neuritis and may lead to an increase in the risk of new episodes. Corticosteroids should not be used in active ocular herpes simplex. |
| Capecitabine | 125 (16.4%) | Not common |
| Acetaminophen | 78 (10.2%) | Not common |
| Lansoprazole | 72 (9.4%) | Not common |
| Ondansetron | 70 (9.2%) | Cases of transient blindness, predominantly during intravenous administration, have been reported. These cases of transient blindness were reported to resolve within a few minutes up to 48 hours. |
| Hydrocortisone | 65 (8.5%) | Not common |
| Levetiracetam | 60 (7.9%) | Not common |
| Chlorpheniramine Maleate | 56 (7.3%) | Not common |
| Cyclizine | 56 (7.3%) | Not common |

**Table S4.** Global assessment through adapted Bradford Hill Criteria.

| **Criteria** | **Description** | **Source/Method** |
| --- | --- | --- |
| Strength of the association | The strength of the disproportionality suggests a robust signal | Disproportionality analysis |
| Analogy | The association was also demonstrated for other anticancer drugs, such as trastuzumab. | Literature search and labels |
| Biological plausibility/  empirical evidence | Off-target toxicity may arise from the cytotoxic payload, whereas on-target toxicity occurs when the drug interacts with its intended receptor, such as HER2, TF, and Nectin-4 proteins expressed in ocular tissues. | Literature search |
| Consistency | Our previous research findings, along with published observational studies, suggest a potential association between ocular toxicity and ADCs. | Disproportionality analysis and literature search |
| Specificity | Pharmacovigilance data suggest a stronger association between ADCs and ocular AEs compared to other drugs. Furthermore, a drug-specific effect, rather than a class-effect, cannot be ruled out. | Disproportionality analysis |
| Temporal relationship | Time-to-onset analysis indicated a temporal relationship between ADC treatment and the occurrence of ocular AEs. | Time-to-onset analysis |
| Reversibility | This criterion has limited applicability in this context due to the absence of data on rechallenge and de-challenge in the FAERS database. | Not applicable |
| Coherence | Randomized controlled trials and case reports support the potential association of ocular toxicity with ADCs. | Literature search |


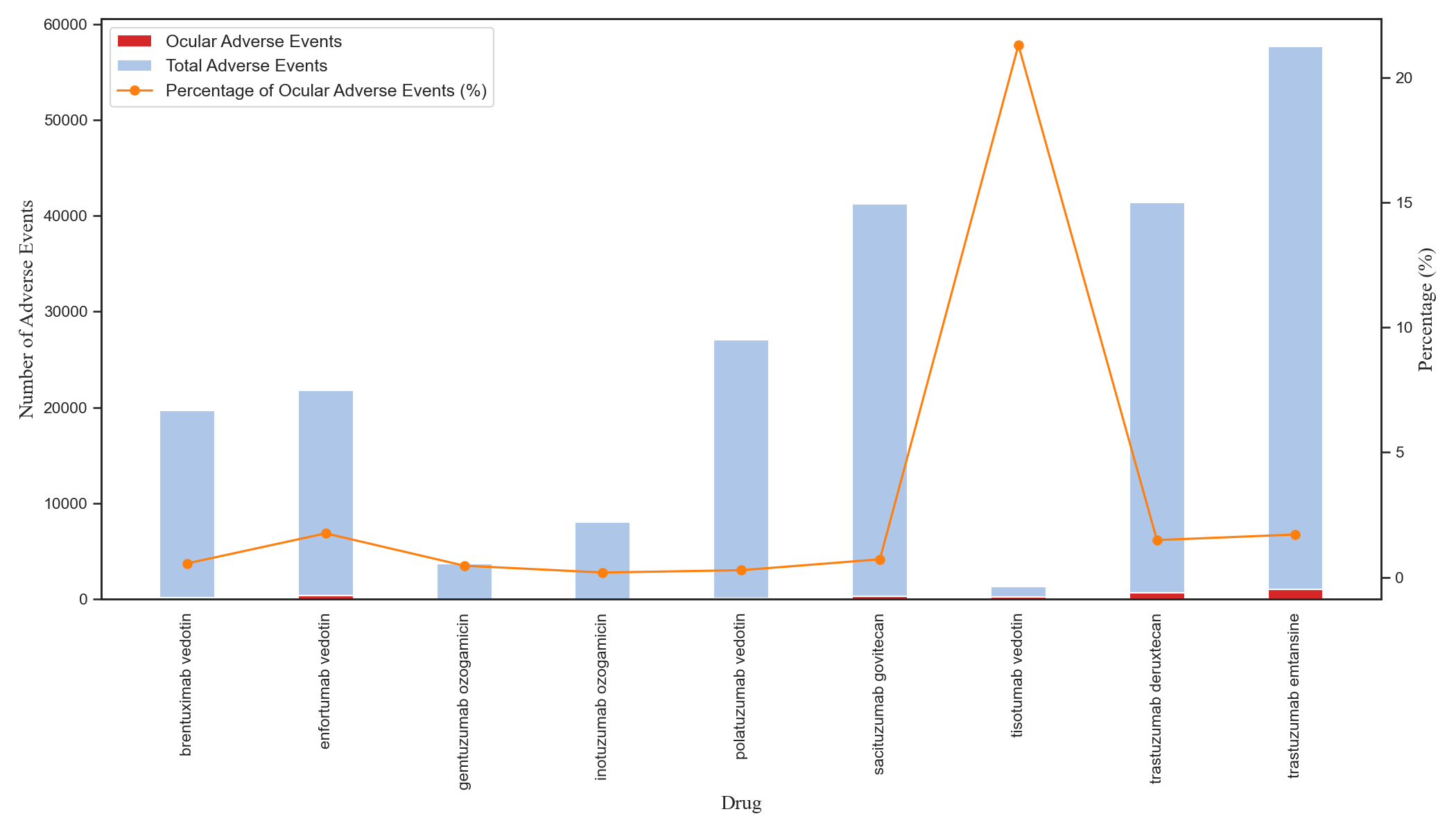


**Figure S1.** The proportion of ocular adverse events with antibody-drug conjugates.


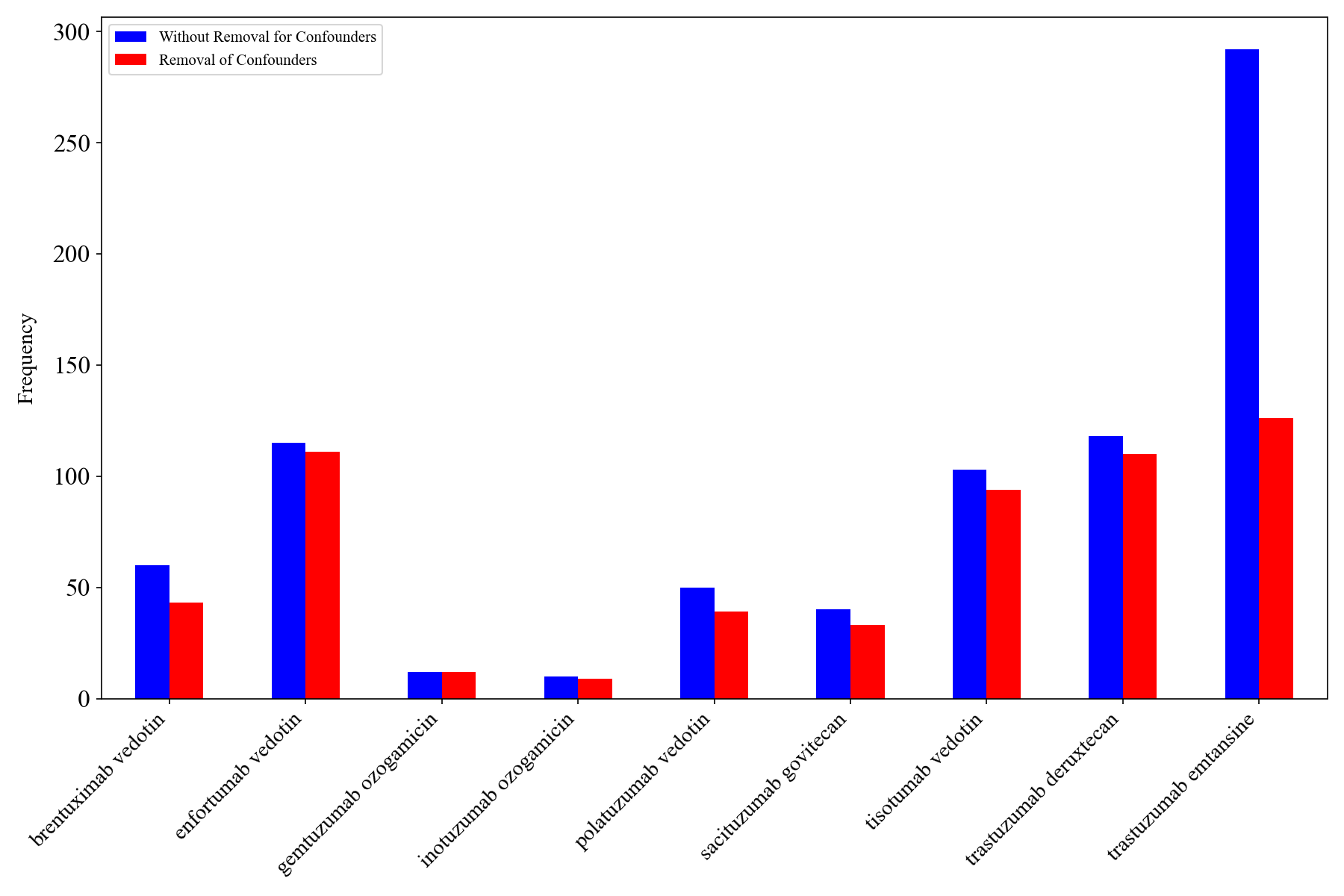


**Figure S2.** Number of cases of ocular AEs with ADCs before and after removal of cases of concomitant drugs.


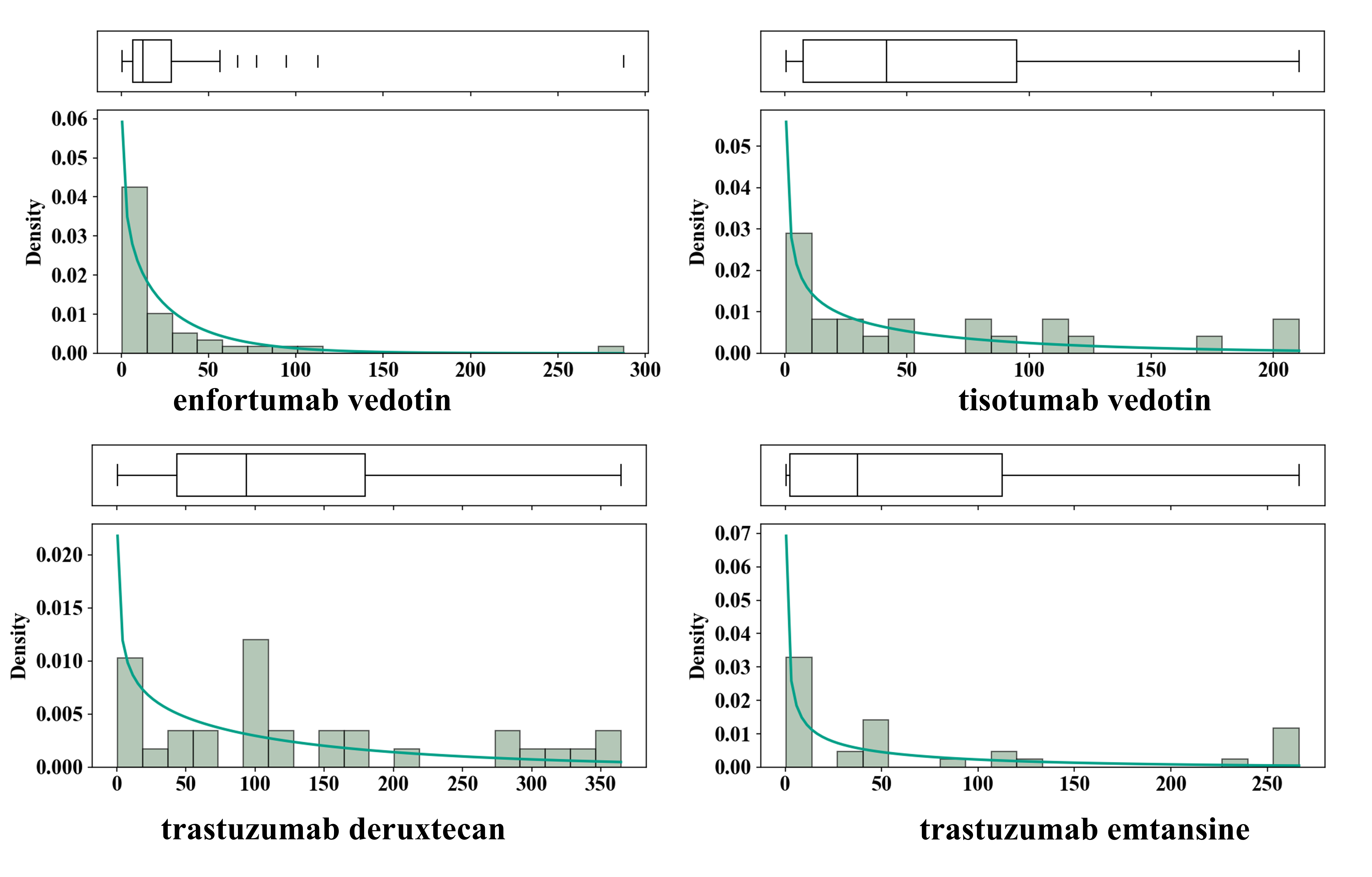


**Figure S3.** TTO analysis of ocular adverse events associated with ADCs.
